# Trends and Significance of VRE Colonization in the ICU: A Meta-Analysis of Published Studies

**DOI:** 10.1371/journal.pone.0075658

**Published:** 2013-09-27

**Authors:** Panayiotis D. Ziakas, Rachana Thapa, Louis B. Rice, Eleftherios Mylonakis

**Affiliations:** 1 Infectious Diseases Division, Rhode Island Hospital and Warren Alpert Medical School of Brown University, Providence, Rhode Island, United States of America; 2 Department of Medicine, Warren Alpert Medical School of Brown University, Providence, Rhode Island, United States of America; Columbia University, United States of America

## Abstract

**Background:**

The burden and significance of vancomycin-resistant enterococci (VRE) colonization in the ICU is not clearly understood.

**Methods:**

We searched PubMed and EMBASE up to May 2013 for studies reporting the prevalence of VRE upon admission to the ICU and performed a meta-analysis to assess rates and trends of VRE colonization. We calculated the prevalence of VRE on admission and the acquisition (colonization and/or infection) rates to estimate time trends and the impact of colonization on ensuing VRE infections.

**Findings:**

Across 37 studies (62,959 patients at risk), the estimated prevalence of VRE on admission to the ICU was 8.8% (7.1-10.6). Estimates were more consistent when cultures were obtained within 24 hours from admission. The VRE acquisition rate was 8.8% (95% CI 6.9-11.0) across 26 evaluable studies (35,364 patients at risk). Across US studies, VRE acquisition rate was 10.2% (95% CI 7.7-13.0) and demonstrated significant decline in annual trends. We used the US estimate of colonization on admission [12.3% (10.5-14.3)] to evaluate the impact of VRE colonization on admission in overall VRE prevalence. We demonstrated that VRE colonization on admission is a major determinant of the overall VRE burden in the ICU. Importantly, among colonized patients (including admitted and/or acquired cases) the VRE infection rates vary widely from 0-45% (with the risk of VRE bacteremia being reported from 0-16%) and <2% among those without a proven colonization.

**Conclusion:**

In summary, up to 10.6% of patients admitted in the ICU are colonized with VRE on admission and a similar percentage will acquire VRE during their ICU stay. Importantly, colonization on admission is a major determinant of VRE dynamics in the ICU and the risk of VRE-related infections is close related to colonization.

## Introduction

The emergence of drug-resistant bacteria in the ICU has been well documented [1-3]. Among these drug resistant pathogens, vancomycin-resistant enterococci (VRE) account for significant excess morbidity and costs [4-6]. Interestingly, VRE is uncommon (<1%) among otherwise healthy individuals [7,8], but it is present in most ICUs. Moreover, ICUs are major reservoirs of VRE that sustain VRE presence in the health care setting [9]. Although the impact of VRE infection in the ICU is significant, the association between VRE colonization and infection has not been established [10,11]. The rationale for conducting this study was to assess the magnitude and significance of VRE colonization at admission in the ICU, as well as its impact in VRE infections.

## Materials and Methods

### Study Selection

MEDLINE and EMBASE were searched for studies in English providing data in VRE rectal colonization upon admission in the ICU. Applied search terms were “VRE OR (vancomycin AND resistant AND enterococ*) AND (ICU OR (critically AND ill) OR (intensive AND care))”. Last access was on May 17, 2013. Relevance to the topic was initially assessed by title and abstract reading. Pertinent articles were accessed in full text to determine eligibility and extract data. The reference lists of eligible studies were screened for additional articles. The PRISMA guidelines were followed (Appendix S1) [12].

Studies that had extractable data on VRE rectal colonization upon admission in the ICU were included in the meta-analysis. Neonatal and pediatric ICUs were excluded from analysis. Abstracts, conference proceedings, and unpublished material were not considered.

### Outcomes of interest

We defined three outcomes of interest: (a). The prevalence of VRE colonization upon admission. (b) Acquisition rates, calculated as the proportion of VRE negative patients at ICU admission that acquire VRE colonization/infection during ICU stay. If the denominator was not provided, it was approximated by subtracting the number of patients colonized with VRE at admission from the total number of patients at risk. (c) The association of admission VRE colonization with ensuing VRE associated infections in the ICU [13,14].

### Data Extraction

Two authors (PZ, RT) independently retrieved and extracted data and consensus was reached in cases of discrepancies. For each study apart from prevalence, we extracted the period of recruitment, country of origin, time lapsed from admission to screening and ICU type, and predominant VRE resistance phenotype.

We extrapolated stratified data by ICU type, calendar year and intervention. If stratified data were not reported, we used the aggregated data. We crudely adjusted for any time-trends by using the mid-year of study recruitment as index year of the study. Date of study publication was not used, because it is not consistent to the time that the study was actually conducted.

### Quality assessment

Studies were given quality points based on items regarding optimal research design and quality of reporting as previously described for schizophrenia [15]. The modified chart with assigned scores is provided with the appendix S2. Studies with quality scores above the 75^th^ percentile were considered of higher quality.

### Data Analysis

We performed a meta-analysis of random-effects (RE) to estimate the pooled (combined) prevalence and 95% confidence intervals, using the Freeman-Tukey arcsine methodology to address stabilizing variances [16]. Der-Simonian & Laird weights were applied [17]. We used the between-study variance τ^2^ to measure statistical heterogeneity [17,18]. Small study effects were addressed with Egger’s test for publication bias [19]. The “trim & fill” method was used to adjust effects for theoretically missing studies [20]. We incorporated a subgroup and meta-regression technique to adjust for potential sources of heterogeneity. For time trends, model coefficients were transformed to rates and fitted values were plotted against the index year along with observed prevalence rates.

VRE prevalence estimates from U.S.A. studies were used to simulate an approximation of VRE endemic burden in the ICU. Assuming that colonized VRE patients remain colonized throughout their length of stay [21,22] and represent the major source of VRE transmission in the ICU, then the predicted endemic prevalence of VRE y_p_ is derived by solving the following equation: R(p,q)= [y_p_ (1+δφ)-(δ+1)φ]/[y_p_(1-y_p_)] [23]. For any given δ, φ, R(p,q), the solution is a quadratic equation with two solutions (one negative and one positive y_p_) when the discriminant (D) is D>0. Only the positive solution applies to our simulation. R(p,q) represents the effective reproductive number for an ICU, δ the proportional increase in length of stay for colonized patients and the φ admission prevalence. The effective reproductive number is a core parameter in infectious disease dynamics within an ICU and it measures the performance of infection control measures. If it is lower than unit, that is R(p,q)<1, transmission alone is unable to sustain VRE endemic in the ICU. That implies that even in ICUs with very low effective reproductive number, i.e R(p,q)<<1, which suggests highly effective preventive measures, introduction of VRE colonized patients will remain the main factor to sustain VRE transmission, and φ will be the lower boundary of endemic prevalence [23]. VRE colonization dynamics were simulated for R=0.9 to approximate the outmost boundary to control VRE endemic in ICU and R=0.5 a modest scenario for an effective restriction policy in the ICU.

The Stata version 11 software package (Stata Corporation, College Station, TX) and StatsDirect version 2.7.9 (StatsDirect Ltd, UK) were used for data analysis.

## Results

Initial search yielded a total of 1,659 potentially relevant publications. A total of 1,569 were excluded on title and abstract reading, leaving 90 studies for full-text evaluation. Thirty-eight studies were deemed appropriate for analysis, of which one was linked to another due to overlapping data [24,25]. Manual search of references lists did not add any additional publications, leaving 37 studies eligible for final analysis (coded from 38 published manuscripts that included 62,959 patients at risk) (Appendix S3-Flow diagram). The summary of studies included in our analysis is presented in Table 1 [10,11,24-59]. VRE colonization rates on admission varied widely from 0.1-42.6% (median 8.7%), and the population at risk from 47 to 8203 patients (median 662 patients). The majority of studies (21/37, 57%) originated in the US, followed in descending order by studies originating in Asia (6/37, 16%), Europe (4/37, 11%), Oceania (3/37, 8%) and S. America (3/37, 8%). Time to VRE screening was up to 24h in 17 studies, up to 48h in 14 studies, and up to 72h or longer in 5 studies. In two studies surveillance screening was performed within the first week of admission (Table 1). Cultures were used for surveillance screening, with the exception of a single study [37] where PCR was used.

**Table 1:** Summary of included studies.

**ID**	**Author**	**Year**	**Origin**	**Mid-year**	**ICU type**	**tScreen/site (detection)**	**VRE (%**)** Ad.** (**N**)	**VRE(%) Acq, (n)**	**Resistance phenotype**	**Quality Score**
1	Climo MW [26]	2013	USA	2008	6 ICUs	48h/R (NA)			NA	B
#1	(Intervention)†						16.3(3970)	2.4 (3323)	NA	
#2	(Control)						15.1(3842)	3.3 (3262)	NA	
2	Batis tao DW [27]	2012	Brasil	2009	ICU	48h/R(C)	15.0(333)	9.9 (283)	VanC (99%)	A
3	Grabsch EA [28]	2012	Australia			Ad/R,F(C)			VanB(>95%)	A
#1				2009	ICU		6.0(662)			
#2				2010	Liver		4.7(1430)			
#3				2009	ICU		9.1(515)			
#4				2010	Liver		8.7(1196)			
4	Kim YJ [29]	2012	Korea	2010	MICU	48h/R(C)	17.6(1048)	12.3 (864)	VanA(100%)	B
5	Pan SC [30]	2012	Taiwan	2008	SICU	24h/R(C)	5.9(871)	5.7 (820)	NA	B
6	Yoon YK [31]	2012	Korea	2010	MICU,SICU	Ad/R(C)	3.4(4445)		NA	B
7	Huang SS [32]	2011	USA	2004	8 ICUs	Ad/R(NA)	8.0(8203)	2.9 (7806)	NA	B
8	Huskins WC [33]	2011	USA	2006		48h/R(C)			NA	B
#1	(Intervention)††				10 ICUs		16.9(2286)	14.8(2132)		
#2	(Control)				8 ICUs		22.1(1503)	12.9(1356)		
9	Minhas P [34]	2011	USA	2008	NICU	48h/ R(C)	2.5(766)		NA	A
10	Climo MW [35]	2009	USA	2005	6 ICUs	48h/R(C)			NA	B
#1	Soap bathing						9.0 (2670)	2.5(2429)		
#2	Chlorhexidine bathing						8.6 (2650)	1.2(2422)		
11	Song JY [36]	2009	Korea	2007	ICU	48h/R(C)	4.4(780)		VanA(100%)	A
12	Wibbenmeyer L[37]	2009	USA	2007	Burn	48h/R (PCR)	10.5(484)	12.1 (423)	NA	A
13	Drees M [38]	2008	USA	2002	MICU,SICU	48h/R (C)	8.9(1330)	4.1 (1212)	NA	B
14	Lambiase A [39]	2007	Italy	2004	ICU	Ad/F,U,Res (C)	3.7(700)		VanA (77%)	B
18	Littvik AM [40]	2006	Argentina	2003	ICU	Ad/R (C)	8.2(147)	4.4(135)	VanA (93%)	A
15	Peta M [41]	2006	Italy	2003	ICU	24h/R (C)	2.6(509)	9.5 (453)	VanA (100%)	B
16	Shadel BN [10]	2006	USA	1998	MICU	Ad/RF (NA)	9.7(1872)	9.8 (1690)	NA	B
17	Vernon MO [42]	2006	USA	2003	MICU	<72h/ R(C)			NA	A
	Soap and water bath						18.0(362)	20.2(77)		
	Chlorhexidine bath						16.0(462)	7.8(77)		
	Non-medicated cloth						19.0(364)	11.5(69)		
19	Furuno JP [43]	2005	USA	2003	MICU,SICU	48h/R (C)	10.1(2440)		NA	B
20	Harris AD [44]	2004	USA	2002	MICU,SICU	72h/R (C)	10.0(1362)		NA	B
21	Winston LG [45]	2004	USA	2003	ICUs	Twice Wk/R(C)			NA	B
	(Post-switch) ‡						7.4(537)	7.6 (497)		
	(Pre-switch)						8.3(399)	11.5 (366)		
22	Yeh KM [46]	2004	Taiwan	2000	ICUs	Ad/R,F (C)	8.0(4538)		VanA>>VanB	B
23	De Jonge E [47]	2003	Holland	2000	ICU	48h/R,Res (C)			NA	B
#1	(Intervention)‡‡						1.4(432)	1.1 (378)		
#2	(Control)						0.9(436)	1.3 (395)		
24	Ho PL [48]	2003	Hong- Kong	1999	ICUs	Ad/R (C)	0.1(1663)		VanA (one case)	A
25	Martinez JA [49]	2003	USA	1997	MICU	48h/R (C)	18.9(169)	22.6 (137)	NA	B
26	Padiglione AA [11]	2003	Australia	1999	11 ICUs	48h/R (C)	0.6(3086)	1.2 (1992)	VanB (92%)	B
27	Warren DK [50]	2003	USA	2000	MICU	Ad/R (C)	24.5(519)	21.0 (352)	NA	A
28	Gardiner D [51]	2002	USA	1999	MICU	24h/R,U,F (C)	42.6(47)	22.2 (27)	NA	A
29	Puzniak LA [52]	2002	USA	1998	MICU	Ad/R,F (C)	7.0(2631)	7.8 (1684)	NA	B
30	Hendrix CW [53]	2001	USA	1996	MICU,SICU	Ad/R,U, Res (C)	9.4(117)	11.3 (106)	NA	B
31	Marin ME [54]	2001	Argentina	2000	ICU	Ad/R (C)	0.7(136)		NA	A
32	Dan M [55]	1999	Israel	1996	ICU	End of wk/R (C)	9.8(61)	14.5 (55)	VanA(100%)	A
33	Grayson ML [56]	1999	Australia	1997	ICU	72h/R,F (C)	0.7(134)		VanB (one case)	A
34	Ostrowsky BE [57]	1999	USA	1995	SICU	24h/R(C)	12.1(290)	14.1 (78)	VanA(85%)	B
35	Zuckerman RA [58]	1999	USA	1995	SICU	24h/R (C)	6.3(80)	9.8 (51)	NA	A
36	Bonten MJ [24,25]	1998	USA	1995	MICU	48h/R, Res,G (C)	14.3(301)	21.3 (258)	VanA(60%)	A
37	Slaughter S [59]	1996	USA	1995	MICU	72h/R (C)	15.5(181)	29.4 (153)	VanA(76%)	A

Data stratified by year, ICU type and intervention (where available). N,n=evaluable samples, tScreen= time within screening cultures were obtained, Ad= when admitted ,C=culture based, G=groins, F=fecal, R=rectal, Res=respiratory, U=urine, NA=not available, Quality score [A (>9), B≤9]. † Chlorhexidine bathing, †† VRA/MRSA surveillance and expanded barrier precautions, ‡ from ticarcillin-clavulanate to piperacillin-tazobactam, ‡‡ selective digestive tract decontamination

### VRE colonization at ICU admission

**Figure 1:**
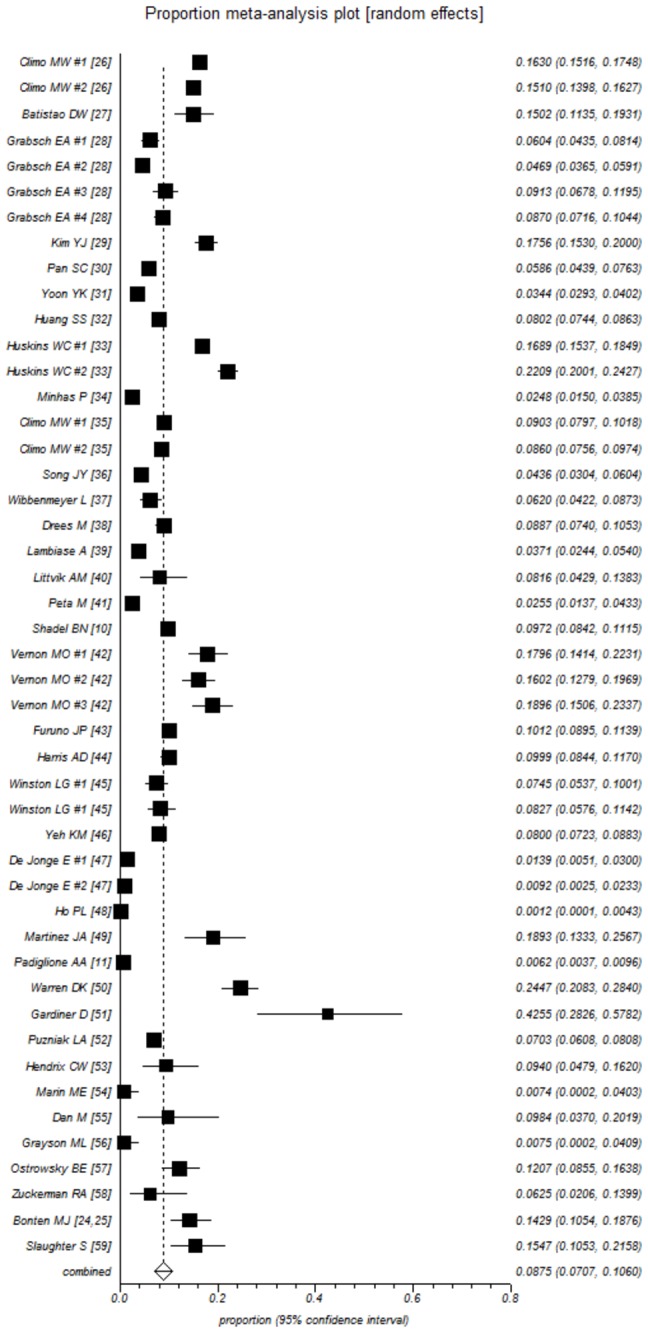
Forrest plot of studies included in the meta-analysis of VRE prevalence at ICU admission [individual study data (squares) and combined estimates (diamond)].

The pooled prevalence estimates are presented in Table 2 and as a forest plot (Figure 1). The estimated prevalence of VRE colonization at ICU admission was 8.8% (95% CI 7.1-10.6). The Egger’s test was insignificant, suggesting absence of small study effects. After excluding studies with observed prevalence rates higher than the 90^th^ percentile (>19%) as potential outliers, the estimated VRE prevalence was 7.9% (95% CI 6.3-9.6). The estimated prevalence across US studies was 12.3% (95% CI 10.5-14.3) and consistent between studies (low between-study variance τ^2^=0.022). US prevalence estimates were higher compared to pooled estimates from European (2.7%, 95% CI 1.3-4.5), Asian (5.3%, 95% CI 2.0-10.2) and Australian (4.4%, 95% 1.5-8.8) studies.

**Table 2:** Summary of Effects.

	Studies (arms)	at risk (N)	Combined Effect (95% CI)	τ^2^
**VRE colonization**				
All studies	37 (47)	62,959	8.8% (7.1-10.6)	0.045
Excluding outliers (>19%)	35 (44)	60,890	7.9% (6.3-9.6).	0.040
USA	21 (27)	39,837	12.3% (10.5-14.3)	0.022
Europe	4 (5)	2,138	2.7% (1.3-4.5)	0.009
Asia	6 (6)	13,345	5.3% (2.0-10.2)	0.051
Australia	3 (6)	7,023	4.4% (1.5-8.8)	0.049
South America	3 (3)	616	7.0 (0.9-18.1)	0.086
Screened up to 24h	17 (20)	30,571	7.3% (5.4-9.4)	0.027
Screened up to 48h	14 (18)	28,526	9.2% (6.2-12.8)	0.060
Screened up to 72h	5 (7)	2,926	12.2% (8.0-17.2)	0.031
**VRE acquisition**				
All studies	26 (33)	35,364	8.8% (6.9-11.0)	0.041
Studies >1000 at risk	8 (11)	29,308	4.8% (2.8-7.2)	0.030
Excluding outliers (>21%)	21 (28)	34,437	7.0%, (5.3-8.9)	0.033
USA	18 (24)	29,989	10.2% (7.7-13.0)	0.042

VRE colonization combined (pooled) estimates.

**Figure 2:**
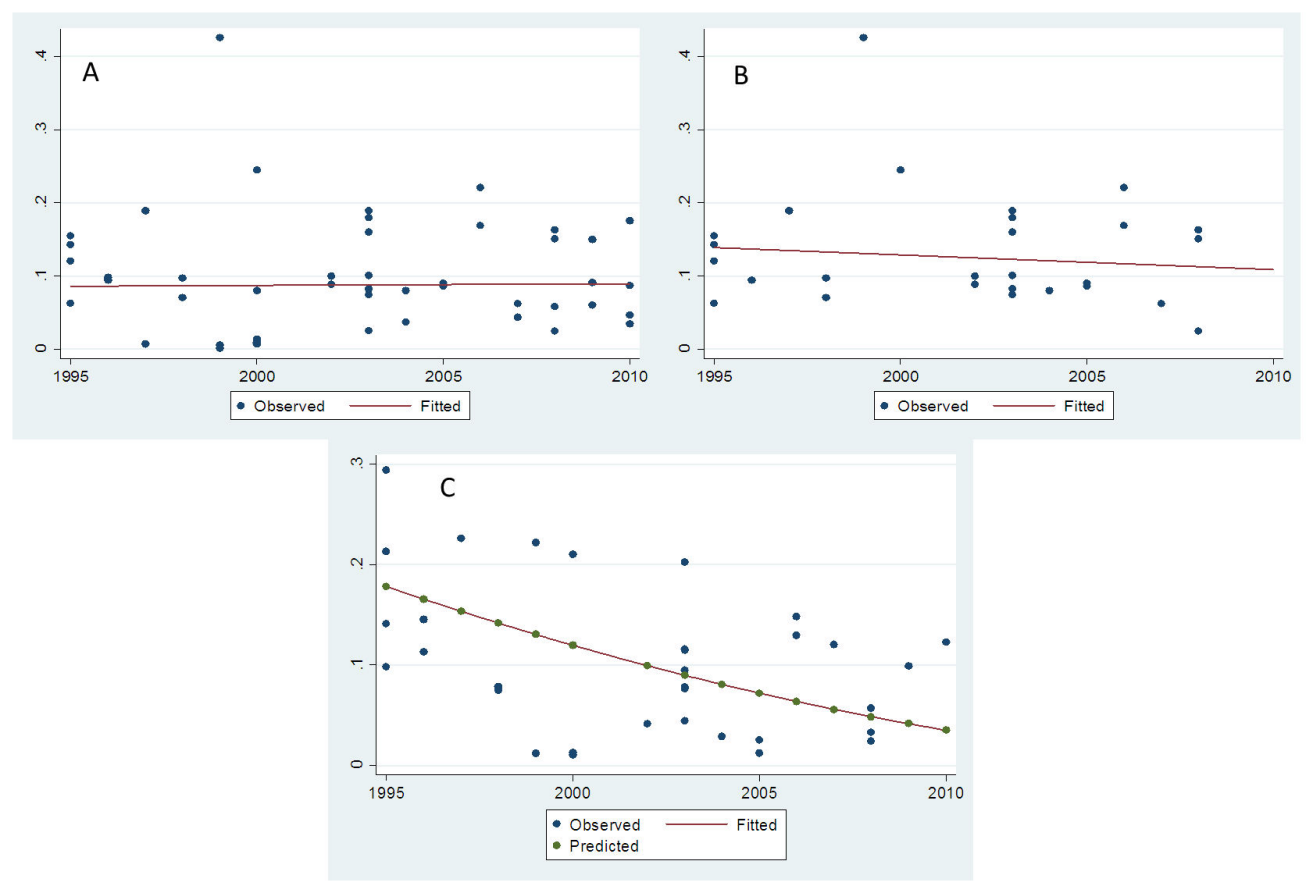
VRE colonization in the ICU. **A**. Observed (dots) and fitted (line) VRE prevalence estimates (all studies), by study mid-year. **B**. Observed (dots) and fitted (line) VRE prevalence estimates (U.S.A. studies), by study mid-year. **C**. Observed (blue dots), predicted (green dots) VRE acquisition estimates and quadratic fit (line) across U.S.A. studies. Data plotted by study mid-year.

Interestingly, time to screening after ICU admission resulted in different VRE prevalence estimates, but these differences did not reach statistical significance. More specifically, rectal surveillance up to 24h after ICU admission yielded an estimated prevalence of 7.3% (95% CI 5.4-9.4). The corresponding estimates for screening up to 48h or ≥72h were 9.2 (95% CI 6.2-12.8) and 12.2 (95% CI 8.0-17.2), respectively. Effects were more consistent (between-study variance τ^2^ =0.027) when screening was performed within 24h of admission. The median quality score was 9 (range 6-10). Studies with higher quality scores (>9) did not differ in the prevalence of VRE (9.3%; 95% 6.0-13.2) from those with lower (≤9) quality scores (8.4%; 95% CI 6.4-10.7). A metaregression analysis was applied to address the effect of time (time trends by mid-year of study recruitment period) on VRE prevalence. No significant effect was noted for all (Figure 2A) or for US studies (Figure 2B), suggesting that on average, VRE admission prevalence has remained stable over 1995 to 2010.

### VRE acquisition during ICU stay

VRE acquisition rates could be extracted in 26 studies with pertinent data. The observed rates varied widely from 1.1% to 29.4% (Table 1; combined effects are presented in Table 2). The estimated VRE acquisition rate was 8.8% (95% CI 6.9-11.0) and estimates did not vary after excluding potential outliers such as studies with observed acquisition rates higher than the 90^th^ percentile, that is >21% (7.0%, 95% CI 5.3-8.9). The Egger’s test was significant, suggesting small study effects (bias 5.8, p=0.002). After excluding studies with <1,000 population at risk, the combined estimate was 4.8% (95% CI 2.8-7.2). As estimates were influenced by smaller studies, a trim-and-fill methodology was used for adjustment, and the estimated risk was 6.7% (95% CI 5.1-8.6).

The estimated acquisition risk across the US studies was 10.2% (95% CI 7.7-13.0). A metaregression analysis was applied to address the effect of time (time trends by index-year) on VRE acquisition across all evaluable studies and US studies only. A decline of marginal significance was noted across all studies (p=0.05). However the decline across US studies was highly significant (p=0.004) and suggested a decline in year-trends for VRE acquisition (Figure 2C).

### VRE colonization and VRE infection during ICU stay

Individual study data could not be pooled because they refer either to prevalent cases at admission or acquired cases or the total number of colonized patients. A descriptive analysis is presented in Table 3. Sixteen studies provided data on VRE infections relative to colonization status. The reported risk of any VRE infection among VRE colonized (prevalent and acquired) ranged from zero [11,27] to a peak of 45% [53]. The reported risk of VRE bacteremia ranged from 0% to 16%. The risk of any VRE infection among non-colonized patients was negligible <2%.

**Table 3:** Descriptive summary of VRE infections by colonization status.

**Author**	**VRE infections**
Batis tao DW,2012 [27]	No VRE infections among prevalent at admission
Pan SC,2012 [30]	5/47 (11%) had VRE infection among acquired cases
Kim YJ, 2012 [29]	28/184 (15%) had VRE infections among those prevalent at admission
Climo MW, 2009 [35]	16/270 (6%) among colonized in soap bathing group had bacteremia (cumulative)
	4/226 (2%) among colonized in chlorhexidine group had bacteremia (cumulative)
Wibbenmeyer L,2009 [37]	7/30 (23%) VRE infections among prevalent at admission vs. 0/463 among VRE negative on admission
	0/51 infections among those acquiring VRE
Lambiase E, 2007 [39]	No VRE bacteremia among prevalent at admission
Peta M, 2006 [41]	2/56 (4%) VRE infections among colonized (cumulative)
Shadel BN, 2006 [10]	8% VRE bacteremia among prevalent at admission vs. <1% among non-colonized
Yeh KM, 2004 [46]	9/816 (1%) had bacteremia among colonized (cumulative)
Littvik AM, 2004 [40]	2/18 (11%) VRE bacteremias among colonized (cumulative)
Martinez JA, 2003 [49]	1/32 (3%) with VRE bacteremia among prevalent at admission
	1/31 (3%) with VRE bacteremia after VRE acquisition
	None among those without VRE colonization
Padiglione AA, 2003 [11]	No VRE infections among colonized (cumulative)
Hendrix CW, 2001 [53]	9/20 (45%) VRE infections among colonized (cumulative)
	0/94 among non-colonized
Dan M, 1999 [55]	1/6 (16%) with VRE bacteremia among prevalent at admission
	1/55 (2%) with VRE bacteremia among not colonized
Ostrowsky BE, 1999 [57]	1/35 (3%) with VRE infection among prevalent at admission
Zuckerman RA, 1999 [58]	No VRE infections among prevalent at admission

### VRE resistance phenotypes

Resistance phenotypes were underreported. Only 15 studies had relevant information to be extracted. Eleven studies reported VanA as the dominant resistance phenotype, while three (all from Australia with a low VRE prevalence) reported a VanB phenotype (Table 1). There was a single study [27] that reported VanC phenotype. The relative lack of information precludes adjusting for the effect of resistance phenotypes.

### VRE admission colonization and VRE prevalence

**Figure 3:**
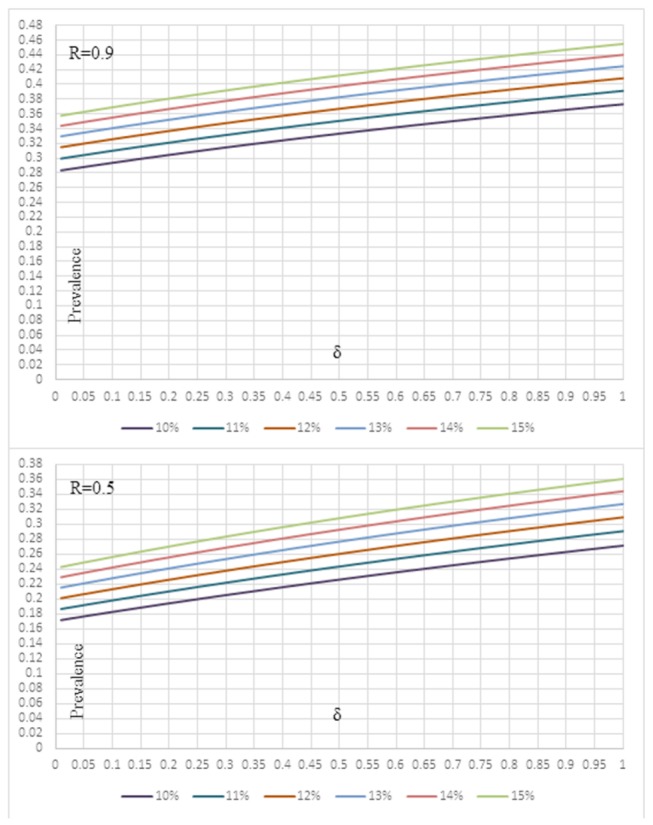
Simulation of VRE endemic prevalence using US admission prevalence estimates to graphically demonstrate the amplifying effect of VRE admission prevalence. **A**. for an effective reproductive number R(p,q)=0.9 B. for an effective reproductive number R(p,q)=0.5.

We also performed a simulation to demonstrate to evaluate how the VRE colonization on admission could affect the overall prevalence of VRE in the ICU. VRE prevalence in the ICU was simulated assuming that preventive measures are of borderline efficacy (that is the effective reproductive number will be just below unit, R=0.9; Figure 3A) or modest efficacy (that is R=0.5; Figure 3B). We used the US estimates for admission colonization (10-15%), a realistic scenario based on our data. Of note is that patients colonized with VRE have a longer ICU stay, compared to non-colonized patients, which increases the risk of transmission [10,36,41,52]. The relative increase of stay is measured by parameter δ in our model, and we adopted a wide range from 0 to 1.0, with 0 representing same length of ICU stay for colonized and non-colonized and 1.0 representing twice the length of stay compared to non-colonized. As shown on Figure 3A and 3B, the predicted estimates suggest not only that admission prevalence defines the lower boundary of VRE colonization, but also that introduction of new cases acts as an amplifier of VRE colonization. In practice, it means that VRE prevalence in an ICU is never expected to be lower than the admission VRE colonization rates and any increase of VRE cases colonized on admission, will add to VRE prevalence in the specific ICU. Therefore, VRE admission rates should be kept low to control VRE endemic potential in the ICU.

## Discussion

We performed a meta-analysis of prevalence rates for VRE colonization at ICU admission and evaluated the significance of VRE colonization on admission to the ICU. Overall, 7.1-10.6% of patients admitted to the ICU are colonized with VRE on admission. Excluding studies with observed rates beyond the 90^th^ percentile as outliers, resulted in a more conservative expectation of VRE colonization at ICU admission (6.3-9.6%). A similar percent (6.9-11.0%) will acquire VRE during their ICU stay (adjusted estimate 5.1-8.6%, after accounting for missing studies). We also found that VRE screening within the first 24 hours of submission gives the most consistent estimate of VRE colonization, compared to studies screening patients within 48h or 72h after ICU admission. The VRE infection rates ranged from 0-45% among colonized patients, while the risk of VRE infection among non-colonized was consistently <2%. Interestingly, as seen in the simulation examples, we demonstrated how VRE colonization on admission to the ICU will determine, in a large degree, the VRE prevalence in the ICU.

The estimate of VRE colonization on admission to the ICU was higher across US studies (12.3%), compared to studies from Europe (2.7%), S. America (7.0%), Asia (5.3%) and Oceania (4.4%). Geographic variations are not unusual for drug-resistant bacteria, as different antibiotic consumption policies and compliance with isolation practices, infection control and antibiotic stewardship programs and cultural differences that affect behavior among the health care personnel, account for this differences [60,61]. Moreover, studies in molecular epidemiology have shown that the worldwide emergence of VRE is related to a specific *Enterococcus faecium* subpopulation CC17. This clade emerged in the U.S., and is likely a major reason why the results in the US are different than elsewhere [62]. Indeed, previous surveillance data have shown that Europe had a lower incidence of VRE [9,63,64]. Interestingly, surveillance data from Europe indicate that the VRE rate might be rising [63,65,66], but ICU data are limited. In analysis, the rate of VRE at admission in the ICU appears to be stable and, since the data from outside the US are sparse, trends and differences between continents should be interpreted with caution. Here it should be noted that as a meta-analysis this study is limited by the quality of included studies, while the exclusion of non-English literature might also expose this analysis to additional bias.

Although as noted above our data showed that the VRE colonization on admission to the ICU has remained stable, the acquisition rates in the US have declined significantly from 1995 to 2010. A plausible explanation is that colonization on admission mostly depends upon factors that can be marginally modified. Such factors include advanced age, prolonged hospitalization, debilitating illness, prior exposure to antibiotics or transfer from other institutions [67]. However, VRE transmission can be prevented by restriction measures and the expanding knowledge in recognition and prevention of VRE infections in the ICU may have contributed for the longitudinal decline in acquisition rates [2]. This analysis indicates that strict control efforts throughout the healthcare system can reduce admission prevalence and contribute significantly to the control of VRE burden within the ICU. It should be emphasized that screening and surveillance for VRE constitute only a part of infection control programs with policies varying across countries and institutions, and their relative impact in overall control cannot be objectively estimated.

Interestingly, we found considerable diversity in the impact of VRE colonization on ensuing VRE-related infections among studies. Overall, our findings indicate that the risk of VRE infection, is not negligible and closely associated to colonization. More specifically, the risk of VRE infection in the ICU among colonized patients varied from 0-45%, while the risk of VRE infection among non-colonized cases was <2%. Of note is that the range of infection among colonized patients varies widely and depends on host factors and population heterogeneity and differences in VRE epidemiology account for these differences. For example, the risk of VRE infection is relatively low in the general hospital setting and in a US study it was 4% [68], but it is higher for high risk populations, such as solid organ transplant recipients (11.3%) [69], cancer patients (29.3%) [70] and allogeneic hematopoietic stem cell transplant recipients (34.2%) [71]. In addition to host factors, the type and virulence of the VRE strain might also influence the risk of VRE infection. For example, studies that focused on the colonization by the *vanC* genotype of *E. faecium* reported no VRE infections [27] or <1% incidence of VRE bacteremia (9-year study among bone marrow transplant recipients) [72].

In conclusion, VRE prevalence on admission to the ICU varied based on geographic location and local epidemiology, but there is no evidence of significant variation over the years. and the observed decline in acquisition rates in the ICU is encouraging. However, the risk of VRE infections it is almost exclusively confined to VRE colonized patients and admission prevalence is the major determinant of VRE dynamics in the ICU. Adequate control of VRE in the ICU can only be achieved by reducing VRE colonization throughout the health system.

## Supporting Information

Appendix S1 PRISMA Checklist S1.
(DOC)

Appendix S2 Study quality data (assigned scores).
(XLSX)

Appendix S3 Flow diagram of meta-analysis.
(DOCX)
