# Supplementary material for: Trends and Significance of VRE Colonization in the ICU: A Meta-Analysis of Published Studies
Source: PLoS One. 2013 Sep 27;8(9):e75658. doi: 10.1371/journal.pone.0075658 (PMC3785502; doi:10.1371/journal.pone.0075658)
Supplement: Appendix S3 — Flow diagram of meta-analysis. (DOCX) [file pone.0075658.s003.docx]

Appendix S3: Flow diagram of meta-analysis

Potentially relevant studies identified and screened for retrieval (N=1659) PubMed n=485, EMBASE n= 1174

Studies excluded Pubmed & EMBASE (n=1569) on title and abstract reading

Non-duplicate studies retrieved for more detailed evaluation (N=90):

Studies excluded (N=52) having no extractable data on VRE colonization and/or admission

- on

Potentially appropriate studies to be included in the meta-analysis (n=38)

one linked to another due to overlapping samples

Studies withdrawn (n=0) Studies added after manual search of references of included studies (n=0)

Studies included in analysis (n=38)

Studies with usable information by outcome: -VRE colonization at ICU admission (n=37) -VRE acquisition during ICU stay (n=26)
